# Tris­(μ_4_-azepane-1-carbodi­thio­ato)bis­(μ_3_-azepane-1-carbodi­thio­ato)-μ_9_-bromido-tetra-μ_2_-bromido-octa­copper(I)­copper(II)

**DOI:** 10.1107/S1600536813009938

**Published:** 2013-04-20

**Authors:** Takashi Okubo, Haruho Anma, Masahiko Maekawa, Takayoshi Kuroda-Sowa

**Affiliations:** a Department of Chemistry, Faculty of Science and Engineering, Kinki University, Higashi-Osaka, Osaka 577-8502, Japan; b PRESTO, Japan Science and Technology Agency (JST), Japan

## Abstract

The reaction of Cu(Hm-dtc)_2_ (H_2_m-dtc is azepane-1-carbodi­thioic acid), CuBr_2_ and methyl iso­thio­cyanate yielded the title mixed-valence nona­nuclear Cu^I^/Cu^II^ compound, [Cu_9_Br_5_(C_7_H_12_NS_2_)_5_] or [Cu^I^_8_Cu^II^Br_5_(Hm-dtc)_5_], encapsulating a bromide anion in the center of the Cu_9_Br_4_S_10_ cluster cage. The cage consists of a mononuclear Cu^II^ unit [Cu(Hm-dtc)_2_], three μ_4_-bridging Hm-dtc^−^ ligands, eight Cu^I^ ions with distorted tetra­hedral or trigonal pyramidal coordination geometries and four μ_2_-bridging bromide anions. The incorporated central bromide anion inter­acts with nine Cu ions with shorter Cu—Br separations than the sum of the van der Waals radii for Cu and Br.

## Related literature

For copper clusters with di­thio­carbamate ligands, see: Cardell *et al.* (2006 ▶); Okubo, Kuwamoto *et al.* (2011 ▶); Liao *et al.* (2012 ▶). For coordination polymers with di­thio­carbamate ligands, see: Golding *et al.* (1974 ▶); Hendrickson *et al.* (1975 ▶); Okubo *et al.* (2010 ▶); Okubo, Tanaka *et al.* (2011 ▶). For pmononuclear copper complexes with di­thio­carbamate ligands, see: Jian *et al.* (1999 ▶); Ngo *et al.* (2003 ▶).
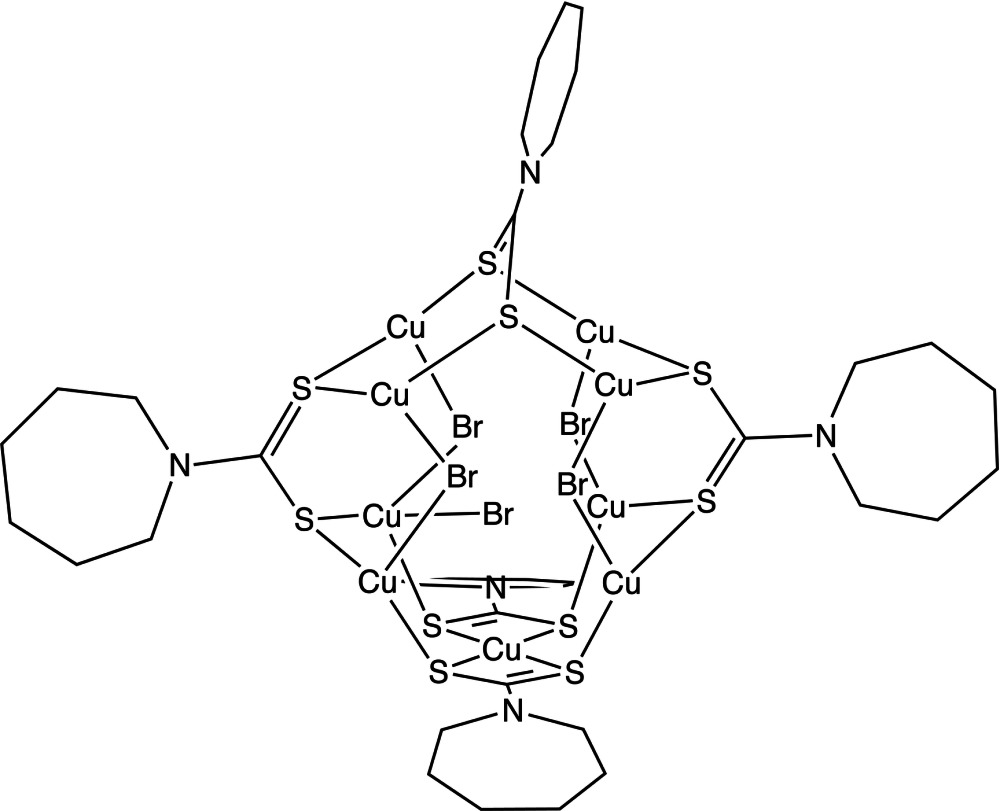


## Experimental

### 

#### Crystal data

[Cu_9_Br_5_(C_7_H_12_NS_2_)_5_]
*M* *_r_* = 1842.93
Monoclinic, 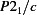

*a* = 12.5728 (6) Å
*b* = 19.5997 (7) Å
*c* = 22.9708 (8) Å
β = 107.0411 (12)°
*V* = 5412.0 (4) Å^3^
*Z* = 4
Mo *K*α radiation
μ = 7.59 mm^−1^
*T* = 296 K
0.90 × 0.60 × 0.10 mm

#### Data collection

Rigaku R-AXIS RAPID diffractometer
Absorption correction: multi-scan (*ABSCOR*; Rigaku, 1995 ▶) *T*_min_ = 0.241, *T*_max_ = 0.468
50620 measured reflections
12284 independent reflections
10468 reflections with *I* > 2σ(*I*)
*R*_int_ = 0.078

#### Refinement

*R*[*F* ^2^ > 2σ(*F* ^2^)] = 0.050
*wR*(*F* ^2^) = 0.122
*S* = 1.03
12284 reflections
577 parameters
H-atom parameters constrained
Δρ_max_ = 2.35 e Å^−3^
Δρ_min_ = −1.54 e Å^−3^

### 

Data collection: *RAPID-AUTO* (Rigaku, 2006 ▶); cell refinement: *RAPID-AUTO*; data reduction: *RAPID-AUTO*; program(s) used to solve structure: *SHELXS97* (Sheldrick, 2008 ▶); program(s) used to refine structure: *SHELXL97* (Sheldrick, 2008 ▶); molecular graphics: *CrystalStructure* (Rigaku, 2010 ▶); software used to prepare material for publication: *CrystalStructure*.

## Supplementary Material

Crystal structure: contains datablock(s) global, I. DOI: 10.1107/S1600536813009938/is5263sup1.cif

Structure factors: contains datablock(s) I. DOI: 10.1107/S1600536813009938/is5263Isup2.hkl

Supplementary material file. DOI: 10.1107/S1600536813009938/is5263Isup4.cdx

Additional supplementary materials:  crystallographic information; 3D view; checkCIF report

## Figures and Tables

**Table 1:** Selected bond lengths (Å)

Br1—Cu3	2.6912 (8)
Br2—Cu2	2.3748 (7)
Br2—Cu6	2.4201 (6)
Br3—Cu3	2.4149 (6)
Br3—Cu7	2.3993 (7)
Br4—Cu5	2.3748 (7)
Br4—Cu9	2.4414 (7)
Br5—Cu4	2.3705 (7)
Br5—Cu8	2.3962 (6)
Cu1—S1	2.3087 (10)
Cu1—S2	2.3208 (13)
Cu1—S3	2.3122 (10)
Cu1—S4	2.3322 (13)
Cu2—S1	2.2805 (12)
Cu2—S5	2.2623 (14)
Cu3—S2	2.3216 (11)
Cu3—S7	2.2711 (10)
Cu4—S3	2.2645 (11)
Cu4—S7	2.2619 (14)
Cu5—S4	2.3110 (12)
Cu5—S5	2.2447 (10)
Cu6—S6	2.2805 (15)
Cu6—S9	2.2537 (12)
Cu7—S8	2.2480 (11)
Cu7—S9	2.2788 (13)
Cu8—S8	2.3043 (15)
Cu8—S10	2.2647 (13)
Cu9—S6	2.2771 (11)
Cu9—S10	2.2705 (12)

**Fig. 1.**
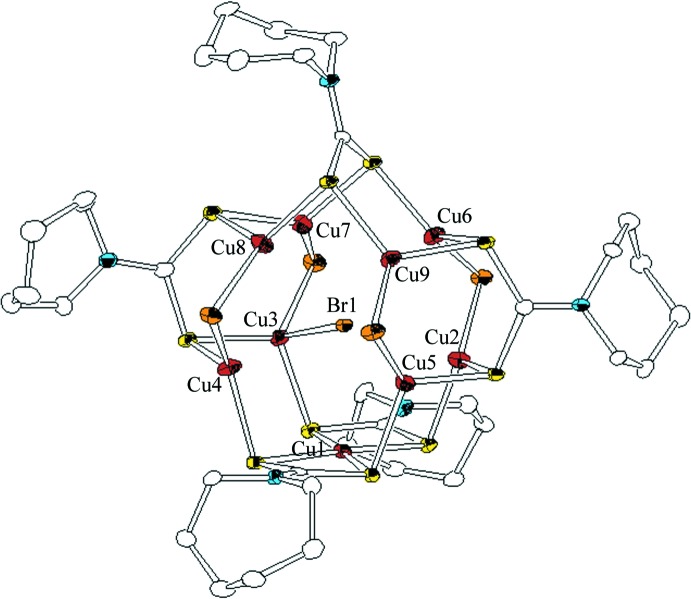
ORTEP view of nonanuclear CuI/CuII cluster with 50% probability level ellipsoids: Cu, red-brown; Br, orange; S, yellow; C, white; and N, blue. Hydrogen atoms are omitted for clarity.

**Fig. 2.**
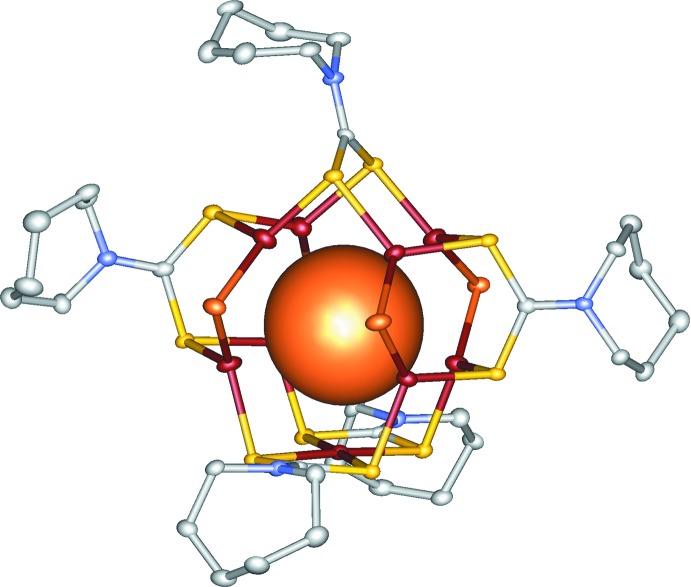
Structure of nonanuclear copper cluster; Cu, red; Br, orange; C, white; S, yellow; N, blue. Hydrogen atoms are omitted for clearly, and incorporated Br1 ion is represented with a space-filling sphere.

**Fig. 3.**
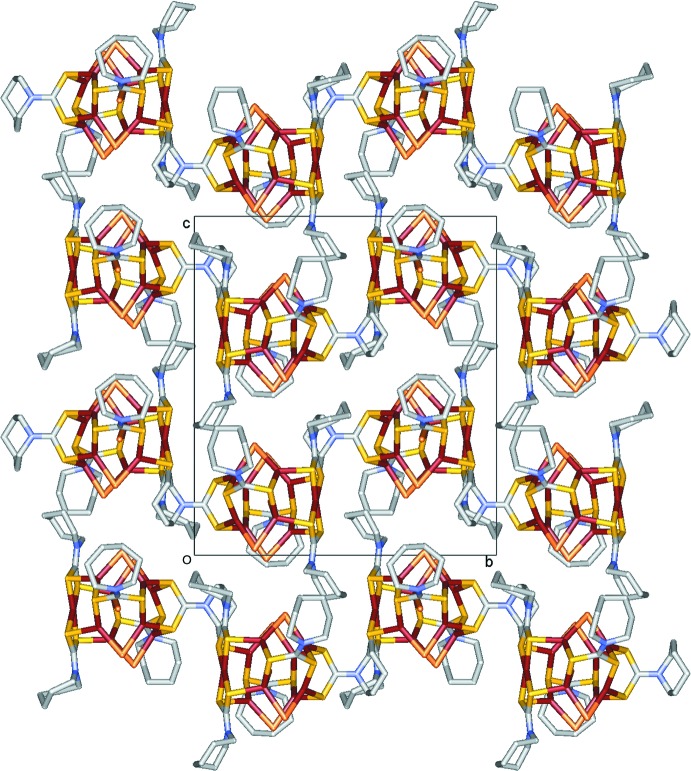
Packing diagram of nonanuclear copper cluster viewed along the a axis. Hydrogen atoms are omitted for clarity.

## supplementary crystallographic information

### Comment

Dithiocarbamate (dtc) derivatives are good candidates for ligands in polynuclear metal complexes. This is because ligands that contain dithiocarboxyl groups have the ability not only to bridge metal ions by sulfur atoms, which have large atomic orbitals, in the ligands but also to stabilize Cu complexes in a wide range of oxidation states such as Cu(I), Cu(II), and Cu(III). To date, several metal clusters (Cardell *et al.*, 2006; Okubo, Kuwamoto *et al.*, 2011; Liao *et al.*, 2012) and coordination polymers (Golding *et al.*, 1974; Hendrickson *et al.*, 1975; Okubo *et al.*, 2010; Okubo, Tanaka *et al.*, 2011) have been synthesized from dithiocarbamate derivatives.

Single-crystal X-ray analysis reveals the formation of a new mixed -valence Cu(I)/Cu(II) cluster of formula [Cu^I^_8_Cu^II^Br_5_(Hm-dtc)_5_]. This complex has a cage structure consisting of a mononuclear Cu(Hm-dtc)_2_ unit, eight Cu ions, four Br anions and bridging µ-Hm-dtc^-^ ligands, and the Br1 is incorporated in the center of the cage through bonding to the Cu3 ion. The Cu1 ion of the mononuclear units has distorted square-planar coordination geometries in which the Hm-dtc^-^ ligands coordinate with the Cu1 ion in four-membered chelate rings. The Cu3 ion forms a tetrahedral S_2_Br_2_ coordination geometry. The other Cu ions, Cu2, Cu4, Cu5, Cu6, Cu7 and Cu8, have trigonal pyramidal S_2_Br_1_ coordination geometries, where the Br1 ion is located close to the Cu ions, thereby forming a pseudo tetrahedral geometry for the Cu ions; the Cu2—Br1, Cu4—Br1, Cu5—Br1, Cu6—Br1, Cu7—Br1, Cu8—Br1 and Cu9—Br1 separations are 2.9054 (6), 2.8672 (6), 2.7825 (8), 2.9319 (6), 2.9262 (8), 2.9563 (7) and 2.9013 (7) Å, respectively; these separations are slightly larger than the Cu3—Br1 distance [2.6912 (8) Å] and smaller than that of the sum of the van der Waals radii for Cu and Br (3.25 Å). In addition, the incorporated Br1 ion is also located close to the Cu1 ion of the mononuclear Cu(Hm-dtc)_2_ unit with the separation of 2.9650 (6) Å. Usually, the oxidation states of Cu complexes with dithiocarbamate ligands can be determined by the Cu—S distances. In the mononuclear Cu(Hm-dtc)_2_ unit, the average Cu—S distance is 2.3185 (13) Å, which is similar to the typical Cu(II)—S distances for Cu(II) -dithiocarbamate complexes such as Cu^II^(Et_2_dtc)_2_ [*av.* 2.312 (1) Å], Cu^II^(*i*-Pr_2_dtc)_2_ [*av.* 2.2884 (7) Å] and Cu^II^(*n*-Bu_2_dtc)_2_ [*av.* 2.308 (1) Å] (Jian *et al.*, 1999; Ngo *et al.*, 2003). Based on its charge neutrality, it is concluded that this complex is in the mixed-valence state with formula [Cu^I^_8_Cu^II^Br_5_(Hm-dtc)_5_], in which the square-planar Cu1 is divalent and the other Cu ions of Cu2—Cu9 with distorted tetrahedral or trigonal pyramidal coordination geometries are monovalent.

### Experimental

A CHCl_3_ solution (20 ml) of Cu(Hm-dtc)_2_ (0.1 mmol) was placed in a 50 ml glass vessel with a screw type cap, and a mixed-solvent (10 ml) of CHCl_3_ and MeOH was slowly added on the solution. Then, a MeOH solution (20 ml) of CuBr_2_ (0.2 mmol) and methylisothiocyanate (1.0 mmol) was slowly added on the solution making the layers of the solutions. By the slow diffusion of the solutions, black plate-shaped single crystals were obtained after a few days standing at room temperature.

### Refinement

All H atoms were placed in calculated positions and refined as riding, with C—H = 0.97 Å and *U*_iso_(H) = 1.2*U*_eq_(C).

### Crystal data

**Table:**

[Cu_9_Br_5_(C_7_H_12_NS_2_)_5_]	*F*(000) = 3604.00
*M**_r_* = 1842.93	*D*_x_ = 2.262 Mg m^−^^3^
Monoclinic, *P*2_1_/*c*	Mo *K*α radiation, λ = 0.71075 Å
Hall symbol: -P 2ybc	Cell parameters from 39225 reflections
*a* = 12.5728 (6) Å	θ = 3.0–27.5°
*b* = 19.5997 (7) Å	µ = 7.59 mm^−^^1^
*c* = 22.9708 (8) Å	*T* = 296 K
β = 107.0411 (12)°	Platelet, black
*V* = 5412.0 (4) Å^3^	0.90 × 0.60 × 0.10 mm
*Z* = 4	

### Data collection

**Table:**

Rigaku R-AXIS RAPID diffractometer	10468 reflections with *F*^2^ > 2.0σ(*F*^2^)
Detector resolution: 10.000 pixels mm^-1^	*R*_int_ = 0.078
ω scans	θ_max_ = 27.4°
Absorption correction: multi-scan (*ABSCOR*; Rigaku, 1995)	*h* = −16→16
*T*_min_ = 0.241, *T*_max_ = 0.468	*k* = −24→25
50620 measured reflections	*l* = −29→29
12284 independent reflections	

### Refinement

**Table:**

Refinement on *F*^2^	Secondary atom site location: difference Fourier map
*R*[*F*^2^ > 2σ(*F*^2^)] = 0.050	Hydrogen site location: inferred from neighbouring sites
*wR*(*F*^2^) = 0.122	H-atom parameters constrained
*S* = 1.03	*w* = 1/[σ^2^(*F*_o_^2^) + (0.0718*P*)^2^] where *P* = (*F*_o_^2^ + 2*F*_c_^2^)/3
12284 reflections	(Δ/σ)_max_ = 0.001
577 parameters	Δρ_max_ = 2.35 e Å^−^^3^
0 restraints	Δρ_min_ = −1.54 e Å^−^^3^
Primary atom site location: structure-invariant direct methods	

### Special details

**Table:**

Geometry. ENTER SPECIAL DETAILS OF THE MOLECULAR GEOMETRY
Refinement. Refinement was performed using all reflections. The weighted *R*-factor (*wR*) and goodness of fit (*S*) are based on *F*^2^. *R*-factor (gt) are based on *F*. The threshold expression of *F*^2^ > 2.0 σ(*F*^2^) is used only for calculating *R*-factor (gt).

### Fractional atomic coordinates and isotropic or equivalent isotropic displacement parameters (Å^2^)

**Table:**

	*x*	*y*	*z*	*U*_iso_*/*U*_eq_	
Br1	0.83480 (3)	0.752744 (17)	0.342115 (17)	0.01264 (10)	
Br2	1.05003 (4)	0.73843 (2)	0.510018 (18)	0.01870 (11)	
Br3	0.71501 (4)	0.70024 (2)	0.480276 (17)	0.01870 (11)	
Br4	0.97088 (4)	0.73887 (2)	0.205891 (19)	0.02023 (11)	
Br5	0.64700 (4)	0.69961 (2)	0.176201 (17)	0.01830 (11)	
Cu1	0.78873 (4)	0.90125 (2)	0.33960 (2)	0.01269 (11)	
Cu2	1.01895 (5)	0.81987 (3)	0.43023 (2)	0.01939 (13)	
Cu3	0.66494 (5)	0.77014 (2)	0.38973 (2)	0.01642 (12)	
Cu4	0.62624 (5)	0.77669 (2)	0.25165 (2)	0.01925 (13)	
Cu5	0.97677 (5)	0.81648 (3)	0.28617 (2)	0.01810 (12)	
Cu6	1.01363 (5)	0.66851 (2)	0.41946 (2)	0.01943 (13)	
Cu7	0.74807 (5)	0.63882 (3)	0.39690 (2)	0.01997 (13)	
Cu8	0.72960 (5)	0.63493 (3)	0.26670 (2)	0.02182 (13)	
Cu9	0.98315 (5)	0.66960 (2)	0.29596 (2)	0.01854 (12)	
S1	0.92264 (9)	0.91814 (5)	0.43167 (4)	0.01244 (19)	
S2	0.68894 (9)	0.88625 (5)	0.40913 (4)	0.0141 (2)	
S3	0.65022 (9)	0.89096 (5)	0.24809 (4)	0.0129 (2)	
S4	0.88384 (9)	0.91962 (5)	0.26776 (4)	0.01251 (19)	
S5	1.12696 (9)	0.82925 (4)	0.36730 (4)	0.0144 (2)	
S6	1.14812 (9)	0.67241 (5)	0.37109 (5)	0.0157 (2)	
S7	0.51331 (9)	0.75009 (4)	0.30896 (4)	0.0137 (2)	
S8	0.60532 (9)	0.60369 (5)	0.31834 (5)	0.0171 (2)	
S9	0.90727 (9)	0.57583 (5)	0.41897 (4)	0.0140 (2)	
S10	0.88493 (9)	0.57066 (5)	0.28136 (4)	0.0132 (2)	
N1	0.8337 (3)	0.88817 (16)	0.52277 (14)	0.0139 (7)	
N2	0.7410 (3)	0.89371 (16)	0.15547 (14)	0.0135 (7)	
N3	1.3153 (3)	0.76012 (15)	0.39905 (15)	0.0134 (7)	
N4	0.3988 (3)	0.64004 (16)	0.25959 (15)	0.0171 (8)	
N5	0.8811 (3)	0.46334 (16)	0.35098 (14)	0.0139 (7)	
C1	0.8178 (4)	0.89505 (18)	0.46438 (17)	0.0125 (8)	
C2	0.9448 (4)	0.8977 (2)	0.56747 (18)	0.0186 (9)	
C3	0.9389 (4)	0.9327 (2)	0.62605 (17)	0.0183 (9)	
C4	0.8612 (4)	0.9946 (2)	0.61714 (19)	0.0193 (9)	
C5	0.7404 (4)	0.9761 (2)	0.61338 (18)	0.0194 (9)	
C6	0.6780 (4)	0.9350 (2)	0.55694 (18)	0.0194 (9)	
C7	0.7390 (4)	0.8710 (2)	0.54670 (18)	0.0172 (9)	
C8	0.7573 (4)	0.89973 (17)	0.21465 (17)	0.0120 (8)	
C9	0.6298 (4)	0.88212 (19)	0.11373 (18)	0.0154 (8)	
C10	0.5613 (4)	0.9483 (2)	0.09793 (18)	0.0180 (9)	
C11	0.6279 (4)	1.0095 (2)	0.0873 (2)	0.0235 (10)	
C12	0.7159 (4)	0.9948 (3)	0.0551 (2)	0.0235 (10)	
C13	0.8288 (4)	0.9721 (2)	0.09796 (19)	0.0223 (10)	
C14	0.8315 (4)	0.9021 (2)	0.12698 (17)	0.0160 (9)	
C15	1.2051 (4)	0.75410 (18)	0.38024 (17)	0.0130 (8)	
C16	1.3719 (4)	0.82783 (19)	0.40894 (18)	0.0153 (8)	
C17	1.3890 (4)	0.8540 (2)	0.47292 (19)	0.0198 (9)	
C18	1.4519 (4)	0.8041 (3)	0.52339 (18)	0.0203 (9)	
C19	1.3852 (4)	0.7392 (2)	0.52702 (19)	0.0211 (10)	
C20	1.3974 (4)	0.6808 (2)	0.48579 (19)	0.0203 (9)	
C21	1.3894 (4)	0.70183 (19)	0.42054 (18)	0.0156 (9)	
C22	0.4969 (4)	0.66228 (19)	0.29298 (17)	0.0149 (8)	
C23	0.3016 (4)	0.6857 (2)	0.2356 (2)	0.0216 (9)	
C24	0.2269 (4)	0.6666 (3)	0.1720 (2)	0.0255 (10)	
C25	0.2884 (5)	0.6504 (3)	0.1262 (2)	0.0271 (11)	
C26	0.3245 (5)	0.5751 (3)	0.1259 (2)	0.0278 (11)	
C27	0.4119 (4)	0.5524 (2)	0.18354 (19)	0.0216 (10)	
C28	0.3801 (4)	0.5675 (2)	0.24224 (18)	0.0200 (9)	
C29	0.8906 (4)	0.53089 (18)	0.35076 (17)	0.0126 (8)	
C30	0.8775 (4)	0.4242 (2)	0.40606 (19)	0.0208 (10)	
C31	0.7587 (5)	0.4085 (3)	0.4052 (2)	0.0251 (10)	
C32	0.6872 (4)	0.3695 (2)	0.3490 (2)	0.0251 (10)	
C33	0.6583 (4)	0.4116 (3)	0.2902 (2)	0.0255 (10)	
C34	0.7465 (4)	0.4142 (3)	0.2566 (2)	0.0235 (10)	
C35	0.8653 (4)	0.42096 (19)	0.29607 (18)	0.0173 (9)	
H2A	0.9906	0.9249	0.5490	0.0223*	
H2B	0.9801	0.8535	0.5776	0.0223*	
H3A	1.0133	0.9473	0.6487	0.0219*	
H3B	0.9150	0.8994	0.6508	0.0219*	
H4A	0.8617	1.0179	0.5800	0.0232*	
H4B	0.8898	1.0259	0.6508	0.0232*	
H5A	0.7408	0.9500	0.6493	0.0233*	
H5B	0.6997	1.0179	0.6141	0.0233*	
H6A	0.6060	0.9219	0.5608	0.0233*	
H6B	0.6651	0.9641	0.5214	0.0233*	
H7A	0.7666	0.8465	0.5848	0.0207*	
H7B	0.6874	0.8414	0.5180	0.0207*	
H9A	0.5905	0.8500	0.1321	0.0184*	
H9B	0.6367	0.8618	0.0765	0.0184*	
H10A	0.4997	0.9406	0.0616	0.0216*	
H10B	0.5302	0.9589	0.1309	0.0216*	
H11A	0.6645	1.0301	0.1265	0.0282*	
H11B	0.5763	1.0429	0.0635	0.0282*	
H12A	0.7269	1.0356	0.0337	0.0282*	
H12B	0.6882	0.9594	0.0249	0.0282*	
H13A	0.8828	0.9725	0.0753	0.0268*	
H13B	0.8525	1.0056	0.1303	0.0268*	
H14A	0.8245	0.8672	0.0961	0.0192*	
H14B	0.9025	0.8958	0.1576	0.0192*	
H16A	1.3275	0.8604	0.3801	0.0184*	
H16B	1.4435	0.8239	0.4013	0.0184*	
H17A	1.3169	0.8638	0.4784	0.0238*	
H17B	1.4301	0.8965	0.4777	0.0238*	
H18A	1.5213	0.7911	0.5160	0.0244*	
H18B	1.4700	0.8275	0.5623	0.0244*	
H19A	1.4081	0.7230	0.5688	0.0253*	
H19B	1.3071	0.7513	0.5170	0.0253*	
H20A	1.3400	0.6472	0.4845	0.0243*	
H20B	1.4687	0.6589	0.5036	0.0243*	
H21A	1.4632	0.7133	0.4183	0.0187*	
H21B	1.3627	0.6634	0.3937	0.0187*	
H23A	0.3284	0.7319	0.2343	0.0260*	
H23B	0.2571	0.6852	0.2637	0.0260*	
H24A	0.1825	0.6272	0.1757	0.0306*	
H24B	0.1762	0.7041	0.1565	0.0306*	
H25A	0.3541	0.6791	0.1346	0.0325*	
H25B	0.2410	0.6621	0.0858	0.0325*	
H26A	0.2593	0.5463	0.1199	0.0333*	
H26B	0.3533	0.5680	0.0916	0.0333*	
H27A	0.4814	0.5753	0.1858	0.0259*	
H27B	0.4239	0.5037	0.1811	0.0259*	
H28A	0.3024	0.5563	0.2359	0.0239*	
H28B	0.4243	0.5392	0.2751	0.0239*	
H30A	0.9138	0.4504	0.4422	0.0250*	
H30B	0.9181	0.3818	0.4079	0.0250*	
H31A	0.7217	0.4513	0.4081	0.0301*	
H31B	0.7610	0.3821	0.4413	0.0301*	
H32A	0.7269	0.3288	0.3433	0.0301*	
H32B	0.6187	0.3550	0.3566	0.0301*	
H33A	0.5905	0.3933	0.2626	0.0306*	
H33B	0.6427	0.4580	0.2999	0.0306*	
H34A	0.7404	0.3730	0.2325	0.0282*	
H34B	0.7298	0.4525	0.2286	0.0282*	
H35A	0.8943	0.3757	0.3086	0.0207*	
H35B	0.9092	0.4403	0.2717	0.0207*	

### Atomic displacement parameters (Å^2^)

**Table:**

	*U*^11^	*U*^22^	*U*^33^	*U*^12^	*U*^13^	*U*^23^
Br1	0.0174 (3)	0.00951 (18)	0.01128 (19)	0.00006 (13)	0.00471 (15)	0.00048 (14)
Br2	0.0293 (3)	0.01343 (19)	0.01172 (19)	−0.00048 (15)	0.00343 (17)	−0.00034 (15)
Br3	0.0282 (3)	0.0187 (2)	0.01104 (19)	0.00405 (16)	0.00865 (17)	0.00271 (16)
Br4	0.0350 (3)	0.01384 (19)	0.0145 (2)	−0.00169 (16)	0.01144 (18)	−0.00015 (16)
Br5	0.0282 (3)	0.0159 (2)	0.00980 (19)	0.00432 (16)	0.00411 (17)	0.00000 (15)
Cu1	0.0197 (3)	0.0114 (3)	0.0073 (3)	−0.00001 (18)	0.00432 (19)	0.00032 (18)
Cu2	0.0265 (4)	0.0157 (3)	0.0181 (3)	0.0045 (2)	0.0097 (3)	0.0026 (2)
Cu3	0.0230 (3)	0.0123 (3)	0.0128 (3)	−0.00221 (19)	0.0036 (2)	−0.0005 (2)
Cu4	0.0317 (4)	0.0116 (3)	0.0184 (3)	−0.0037 (2)	0.0134 (3)	−0.0013 (2)
Cu5	0.0231 (4)	0.0148 (3)	0.0150 (3)	0.00341 (19)	0.0032 (2)	0.0006 (2)
Cu6	0.0284 (4)	0.0151 (3)	0.0152 (3)	−0.0063 (2)	0.0071 (3)	−0.0027 (2)
Cu7	0.0246 (4)	0.0201 (3)	0.0144 (3)	0.0040 (2)	0.0045 (3)	−0.0023 (2)
Cu8	0.0275 (4)	0.0225 (3)	0.0154 (3)	0.0082 (3)	0.0061 (3)	0.0050 (3)
Cu9	0.0263 (4)	0.0143 (3)	0.0142 (3)	−0.0048 (2)	0.0046 (3)	0.0009 (2)
S1	0.0184 (6)	0.0112 (4)	0.0087 (4)	−0.0014 (4)	0.0054 (4)	−0.0004 (4)
S2	0.0217 (6)	0.0119 (5)	0.0091 (5)	−0.0024 (4)	0.0053 (4)	−0.0025 (4)
S3	0.0179 (6)	0.0106 (5)	0.0107 (5)	0.0002 (4)	0.0049 (4)	0.0003 (4)
S4	0.0168 (6)	0.0119 (5)	0.0082 (4)	−0.0002 (4)	0.0027 (4)	0.0006 (4)
S5	0.0184 (6)	0.0092 (4)	0.0148 (5)	0.0013 (4)	0.0039 (4)	0.0010 (4)
S6	0.0195 (6)	0.0083 (4)	0.0187 (5)	−0.0003 (4)	0.0046 (4)	−0.0000 (4)
S7	0.0191 (6)	0.0107 (5)	0.0118 (5)	−0.0022 (4)	0.0053 (4)	−0.0016 (4)
S8	0.0222 (6)	0.0112 (5)	0.0164 (5)	0.0003 (4)	0.0033 (4)	−0.0002 (4)
S9	0.0218 (6)	0.0100 (5)	0.0098 (5)	−0.0004 (4)	0.0040 (4)	0.0007 (4)
S10	0.0214 (6)	0.0094 (4)	0.0088 (4)	−0.0008 (4)	0.0045 (4)	0.0006 (4)
N1	0.019 (2)	0.0132 (16)	0.0097 (15)	−0.0025 (13)	0.0047 (14)	−0.0031 (14)
N2	0.020 (2)	0.0120 (15)	0.0074 (15)	−0.0017 (13)	0.0027 (13)	0.0004 (13)
N3	0.019 (2)	0.0095 (15)	0.0112 (16)	0.0008 (13)	0.0035 (14)	0.0007 (13)
N4	0.021 (2)	0.0126 (16)	0.0169 (17)	−0.0010 (14)	0.0039 (15)	0.0018 (14)
N5	0.023 (2)	0.0096 (15)	0.0112 (16)	−0.0014 (13)	0.0076 (14)	0.0015 (13)
C1	0.018 (3)	0.0065 (16)	0.0125 (18)	−0.0024 (14)	0.0045 (16)	0.0000 (15)
C2	0.022 (3)	0.018 (2)	0.0137 (19)	0.0001 (16)	0.0022 (17)	−0.0035 (17)
C3	0.026 (3)	0.0157 (19)	0.0099 (18)	0.0033 (17)	−0.0000 (17)	0.0032 (17)
C4	0.027 (3)	0.0121 (18)	0.017 (2)	−0.0009 (16)	0.0033 (18)	−0.0022 (17)
C5	0.033 (3)	0.0133 (19)	0.0126 (19)	0.0028 (17)	0.0086 (18)	−0.0007 (17)
C6	0.024 (3)	0.021 (2)	0.0142 (19)	−0.0019 (17)	0.0076 (18)	−0.0027 (18)
C7	0.024 (3)	0.019 (2)	0.0106 (18)	−0.0104 (17)	0.0080 (17)	−0.0049 (17)
C8	0.019 (3)	0.0054 (16)	0.0132 (18)	0.0021 (14)	0.0064 (16)	0.0031 (15)
C9	0.022 (3)	0.0118 (18)	0.0116 (18)	−0.0032 (15)	0.0031 (16)	−0.0021 (16)
C10	0.024 (3)	0.0154 (19)	0.0126 (19)	0.0032 (16)	0.0018 (17)	−0.0017 (17)
C11	0.035 (3)	0.015 (2)	0.019 (2)	0.0040 (18)	0.0064 (19)	0.0022 (18)
C12	0.032 (3)	0.018 (2)	0.020 (2)	0.0007 (18)	0.007 (2)	0.0111 (19)
C13	0.029 (3)	0.022 (3)	0.018 (2)	−0.0056 (18)	0.0105 (19)	0.0029 (19)
C14	0.019 (3)	0.019 (2)	0.0111 (18)	−0.0005 (16)	0.0063 (16)	0.0013 (17)
C15	0.020 (3)	0.0127 (18)	0.0073 (17)	0.0001 (15)	0.0059 (16)	−0.0012 (15)
C16	0.017 (3)	0.0118 (18)	0.0164 (19)	−0.0028 (15)	0.0036 (16)	0.0018 (16)
C17	0.019 (3)	0.017 (2)	0.022 (3)	−0.0053 (16)	0.0039 (18)	−0.0028 (18)
C18	0.025 (3)	0.025 (3)	0.0105 (18)	−0.0027 (18)	0.0035 (17)	−0.0032 (18)
C19	0.028 (3)	0.022 (2)	0.013 (2)	−0.0021 (18)	0.0049 (18)	0.0077 (18)
C20	0.023 (3)	0.018 (2)	0.020 (2)	0.0014 (17)	0.0077 (18)	0.0070 (18)
C21	0.019 (3)	0.0129 (18)	0.0137 (19)	0.0032 (15)	0.0026 (16)	0.0030 (16)
C22	0.025 (3)	0.0121 (18)	0.0085 (17)	−0.0014 (16)	0.0067 (16)	0.0002 (16)
C23	0.018 (3)	0.020 (2)	0.023 (3)	0.0019 (17)	0.0006 (18)	−0.0031 (19)
C24	0.022 (3)	0.022 (3)	0.030 (3)	0.0026 (18)	0.004 (2)	−0.001 (2)
C25	0.041 (4)	0.021 (3)	0.020 (3)	0.006 (2)	0.010 (2)	0.008 (2)
C26	0.041 (4)	0.024 (3)	0.018 (3)	−0.002 (2)	0.008 (2)	−0.0019 (19)
C27	0.032 (3)	0.0128 (19)	0.021 (2)	−0.0058 (17)	0.0090 (19)	−0.0019 (18)
C28	0.029 (3)	0.014 (2)	0.0150 (19)	−0.0082 (17)	0.0045 (18)	−0.0005 (17)
C29	0.016 (3)	0.0099 (17)	0.0108 (18)	0.0029 (14)	0.0017 (15)	0.0038 (15)
C30	0.033 (3)	0.0113 (19)	0.018 (2)	−0.0003 (17)	0.0071 (19)	0.0049 (17)
C31	0.041 (4)	0.019 (2)	0.022 (3)	0.0034 (19)	0.019 (2)	0.0047 (19)
C32	0.032 (3)	0.0117 (19)	0.036 (3)	−0.0049 (18)	0.017 (3)	0.003 (2)
C33	0.024 (3)	0.023 (3)	0.031 (3)	−0.0039 (19)	0.011 (2)	−0.006 (2)
C34	0.028 (3)	0.022 (3)	0.021 (2)	−0.0044 (18)	0.009 (2)	−0.0034 (19)
C35	0.026 (3)	0.0103 (18)	0.020 (2)	0.0008 (16)	0.0124 (18)	−0.0024 (17)

### Geometric parameters (Å, º)

**Table:**

Br1—Cu3	2.6912 (8)	C24—C25	1.511 (8)
Br2—Cu2	2.3748 (7)	C25—C26	1.546 (7)
Br2—Cu6	2.4201 (6)	C26—C27	1.518 (6)
Br3—Cu3	2.4149 (6)	C27—C28	1.543 (7)
Br3—Cu7	2.3993 (7)	C30—C31	1.520 (8)
Br4—Cu5	2.3748 (7)	C31—C32	1.544 (6)
Br4—Cu9	2.4414 (7)	C32—C33	1.534 (7)
Br5—Cu4	2.3705 (7)	C33—C34	1.525 (8)
Br5—Cu8	2.3962 (6)	C34—C35	1.510 (6)
Cu1—S1	2.3087 (10)	C2—H2A	0.970
Cu1—S2	2.3208 (13)	C2—H2B	0.970
Cu1—S3	2.3122 (10)	C3—H3A	0.970
Cu1—S4	2.3322 (13)	C3—H3B	0.970
Cu2—S1	2.2805 (12)	C4—H4A	0.970
Cu2—S5	2.2623 (14)	C4—H4B	0.970
Cu3—S2	2.3216 (11)	C5—H5A	0.970
Cu3—S7	2.2711 (10)	C5—H5B	0.970
Cu4—S3	2.2645 (11)	C6—H6A	0.970
Cu4—S7	2.2619 (14)	C6—H6B	0.970
Cu5—S4	2.3110 (12)	C7—H7A	0.970
Cu5—S5	2.2447 (10)	C7—H7B	0.970
Cu6—S6	2.2805 (15)	C9—H9A	0.970
Cu6—S9	2.2537 (12)	C9—H9B	0.970
Cu7—S8	2.2480 (11)	C10—H10A	0.970
Cu7—S9	2.2788 (13)	C10—H10B	0.970
Cu8—S8	2.3043 (15)	C11—H11A	0.970
Cu8—S10	2.2647 (13)	C11—H11B	0.970
Cu9—S6	2.2771 (11)	C12—H12A	0.970
Cu9—S10	2.2705 (12)	C12—H12B	0.970
S1—C1	1.757 (5)	C13—H13A	0.970
S2—C1	1.749 (4)	C13—H13B	0.970
S3—C8	1.744 (5)	C14—H14A	0.970
S4—C8	1.741 (4)	C14—H14B	0.970
S5—C15	1.747 (4)	C16—H16A	0.970
S6—C15	1.741 (4)	C16—H16B	0.970
S7—C22	1.759 (4)	C17—H17A	0.970
S8—C22	1.747 (4)	C17—H17B	0.970
S9—C29	1.756 (4)	C18—H18A	0.970
S10—C29	1.757 (4)	C18—H18B	0.970
N1—C1	1.304 (5)	C19—H19A	0.970
N1—C2	1.483 (5)	C19—H19B	0.970
N1—C7	1.489 (7)	C20—H20A	0.970
N2—C8	1.319 (5)	C20—H20B	0.970
N2—C9	1.462 (5)	C21—H21A	0.970
N2—C14	1.478 (7)	C21—H21B	0.970
N3—C15	1.330 (6)	C23—H23A	0.970
N3—C16	1.491 (5)	C23—H23B	0.970
N3—C21	1.465 (5)	C24—H24A	0.970
N4—C22	1.321 (5)	C24—H24B	0.970
N4—C23	1.484 (6)	C25—H25A	0.970
N4—C28	1.476 (5)	C25—H25B	0.970
N5—C29	1.330 (5)	C26—H26A	0.970
N5—C30	1.492 (6)	C26—H26B	0.970
N5—C35	1.474 (6)	C27—H27A	0.970
C2—C3	1.531 (6)	C27—H27B	0.970
C3—C4	1.532 (6)	C28—H28A	0.970
C4—C5	1.539 (7)	C28—H28B	0.970
C5—C6	1.532 (6)	C30—H30A	0.970
C6—C7	1.524 (7)	C30—H30B	0.970
C9—C10	1.541 (6)	C31—H31A	0.970
C10—C11	1.521 (7)	C31—H31B	0.970
C11—C12	1.530 (8)	C32—H32A	0.970
C12—C13	1.537 (6)	C32—H32B	0.970
C13—C14	1.521 (6)	C33—H33A	0.970
C16—C17	1.511 (6)	C33—H33B	0.970
C17—C18	1.545 (6)	C34—H34A	0.970
C18—C19	1.540 (7)	C34—H34B	0.970
C19—C20	1.522 (7)	C35—H35A	0.970
C20—C21	1.529 (7)	C35—H35B	0.970
C23—C24	1.533 (6)		
			
Br1···Cu1	2.9650 (6)	Br1···Cu6	2.9319 (6)
Br1···Cu2	2.9054 (6)	Br1···Cu7	2.9262 (8)
Br1···Cu4	2.8672 (6)	Br1···Cu8	2.9563 (7)
Br1···Cu5	2.7825 (8)	Br1···Cu9	2.9013 (7)
			
Cu2—Br2—Cu6	76.72 (2)	C5—C4—H4A	108.819
Cu3—Br3—Cu7	70.10 (2)	C5—C4—H4B	108.819
Cu5—Br4—Cu9	73.64 (2)	H4A—C4—H4B	107.701
Cu4—Br5—Cu8	79.37 (2)	C4—C5—H5A	108.513
S1—Cu1—S2	77.52 (4)	C4—C5—H5B	108.501
S1—Cu1—S3	176.43 (5)	C6—C5—H5A	108.516
S1—Cu1—S4	103.80 (5)	C6—C5—H5B	108.508
S2—Cu1—S3	101.51 (5)	H5A—C5—H5B	107.511
S2—Cu1—S4	177.71 (4)	C5—C6—H6A	108.758
S3—Cu1—S4	77.05 (4)	C5—C6—H6B	108.755
Br2—Cu2—S1	121.63 (4)	C7—C6—H6A	108.737
Br2—Cu2—S5	123.64 (4)	C7—C6—H6B	108.758
S1—Cu2—S5	111.18 (5)	H6A—C6—H6B	107.645
Br1—Cu3—Br3	103.92 (3)	N1—C7—H7A	109.362
Br1—Cu3—S2	97.37 (4)	N1—C7—H7B	109.360
Br1—Cu3—S7	102.84 (4)	C6—C7—H7A	109.374
Br3—Cu3—S2	113.85 (3)	C6—C7—H7B	109.362
Br3—Cu3—S7	123.37 (4)	H7A—C7—H7B	108.003
S2—Cu3—S7	110.93 (4)	N2—C9—H9A	109.069
Br5—Cu4—S3	124.02 (4)	N2—C9—H9B	109.070
Br5—Cu4—S7	120.07 (3)	C10—C9—H9A	109.074
S3—Cu4—S7	111.22 (5)	C10—C9—H9B	109.065
Br4—Cu5—S4	121.27 (3)	H9A—C9—H9B	107.827
Br4—Cu5—S5	122.91 (4)	C9—C10—H10A	108.785
S4—Cu5—S5	108.15 (4)	C9—C10—H10B	108.778
Br2—Cu6—S6	114.79 (4)	C11—C10—H10A	108.791
Br2—Cu6—S9	115.04 (4)	C11—C10—H10B	108.785
S6—Cu6—S9	123.24 (5)	H10A—C10—H10B	107.654
Br3—Cu7—S8	120.67 (5)	C10—C11—H11A	108.265
Br3—Cu7—S9	116.14 (3)	C10—C11—H11B	108.266
S8—Cu7—S9	117.08 (5)	C12—C11—H11A	108.266
Br5—Cu8—S8	113.60 (4)	C12—C11—H11B	108.267
Br5—Cu8—S10	123.98 (4)	H11A—C11—H11B	107.374
S8—Cu8—S10	117.45 (5)	C11—C12—H12A	108.747
Br4—Cu9—S6	116.49 (4)	C11—C12—H12B	108.749
Br4—Cu9—S10	116.95 (3)	C13—C12—H12A	108.747
S6—Cu9—S10	117.89 (4)	C13—C12—H12B	108.751
Cu1—S1—Cu2	96.51 (4)	H12A—C12—H12B	107.652
Cu1—S1—C1	85.28 (12)	C12—C13—H13A	108.268
Cu2—S1—C1	104.88 (13)	C12—C13—H13B	108.264
Cu1—S2—Cu3	93.33 (5)	C14—C13—H13A	108.277
Cu1—S2—C1	85.09 (16)	C14—C13—H13B	108.266
Cu3—S2—C1	106.06 (13)	H13A—C13—H13B	107.380
Cu1—S3—Cu4	97.25 (4)	N2—C14—H14A	109.231
Cu1—S3—C8	85.34 (12)	N2—C14—H14B	109.225
Cu4—S3—C8	103.98 (13)	C13—C14—H14A	109.230
Cu1—S4—Cu5	94.13 (4)	C13—C14—H14B	109.240
Cu1—S4—C8	84.77 (16)	H14A—C14—H14B	107.919
Cu5—S4—C8	103.96 (13)	N3—C16—H16A	109.193
Cu2—S5—Cu5	90.29 (5)	N3—C16—H16B	109.197
Cu2—S5—C15	103.63 (16)	C17—C16—H16A	109.191
Cu5—S5—C15	109.97 (13)	C17—C16—H16B	109.193
Cu6—S6—Cu9	74.20 (4)	H16A—C16—H16B	107.907
Cu6—S6—C15	108.03 (17)	C16—C17—H17A	108.709
Cu9—S6—C15	111.40 (13)	C16—C17—H17B	108.701
Cu3—S7—Cu4	85.17 (5)	C18—C17—H17A	108.695
Cu3—S7—C22	111.02 (13)	C18—C17—H17B	108.693
Cu4—S7—C22	99.16 (17)	H17A—C17—H17B	107.617
Cu7—S8—Cu8	80.19 (4)	C17—C18—H18A	108.907
Cu7—S8—C22	115.33 (13)	C17—C18—H18B	108.907
Cu8—S8—C22	103.55 (16)	C19—C18—H18A	108.898
Cu6—S9—Cu7	92.62 (5)	C19—C18—H18B	108.903
Cu6—S9—C29	108.91 (15)	H18A—C18—H18B	107.720
Cu7—S9—C29	101.90 (13)	C18—C19—H19A	108.367
Cu8—S10—Cu9	87.41 (4)	C18—C19—H19B	108.359
Cu8—S10—C29	100.71 (15)	C20—C19—H19A	108.367
Cu9—S10—C29	111.66 (13)	C20—C19—H19B	108.377
C1—N1—C2	121.9 (4)	H19A—C19—H19B	107.437
C1—N1—C7	120.4 (4)	C19—C20—H20A	108.597
C2—N1—C7	117.8 (4)	C19—C20—H20B	108.600
C8—N2—C9	121.3 (4)	C21—C20—H20A	108.602
C8—N2—C14	122.6 (4)	C21—C20—H20B	108.596
C9—N2—C14	116.0 (4)	H20A—C20—H20B	107.574
C15—N3—C16	122.2 (4)	N3—C21—H21A	108.939
C15—N3—C21	122.6 (4)	N3—C21—H21B	108.946
C16—N3—C21	114.5 (4)	C20—C21—H21A	108.952
C22—N4—C23	122.9 (4)	C20—C21—H21B	108.944
C22—N4—C28	121.5 (4)	H21A—C21—H21B	107.761
C23—N4—C28	115.6 (3)	N4—C23—H23A	108.639
C29—N5—C30	122.7 (4)	N4—C23—H23B	108.631
C29—N5—C35	123.2 (4)	C24—C23—H23A	108.634
C30—N5—C35	113.9 (3)	C24—C23—H23B	108.638
S1—C1—S2	111.5 (3)	H23A—C23—H23B	107.575
S1—C1—N1	124.0 (3)	C23—C24—H24A	108.572
S2—C1—N1	124.5 (4)	C23—C24—H24B	108.572
N1—C2—C3	112.8 (4)	C25—C24—H24A	108.571
C2—C3—C4	115.5 (4)	C25—C24—H24B	108.586
C3—C4—C5	113.7 (4)	H24A—C24—H24B	107.546
C4—C5—C6	115.0 (4)	C24—C25—H25A	108.648
C5—C6—C7	114.0 (4)	C24—C25—H25B	108.654
N1—C7—C6	111.3 (4)	C26—C25—H25A	108.645
S3—C8—S4	112.2 (3)	C26—C25—H25B	108.651
S3—C8—N2	122.5 (3)	H25A—C25—H25B	107.601
S4—C8—N2	125.2 (4)	C25—C26—H26A	108.637
N2—C9—C10	112.6 (3)	C25—C26—H26B	108.637
C9—C10—C11	113.9 (4)	C27—C26—H26A	108.636
C10—C11—C12	116.1 (4)	C27—C26—H26B	108.641
C11—C12—C13	114.0 (4)	H26A—C26—H26B	107.586
C12—C13—C14	116.1 (4)	C26—C27—H27A	108.864
N2—C14—C13	111.9 (4)	C26—C27—H27B	108.865
S5—C15—S6	124.3 (3)	C28—C27—H27A	108.874
S5—C15—N3	117.4 (3)	C28—C27—H27B	108.863
S6—C15—N3	118.2 (3)	H27A—C27—H27B	107.713
N3—C16—C17	112.1 (4)	N4—C28—H28A	109.421
C16—C17—C18	114.2 (4)	N4—C28—H28B	109.410
C17—C18—C19	113.4 (4)	C27—C28—H28A	109.439
C18—C19—C20	115.7 (5)	C27—C28—H28B	109.430
C19—C20—C21	114.7 (4)	H28A—C28—H28B	108.022
N3—C21—C20	113.2 (4)	N5—C30—H30A	109.320
S7—C22—S8	122.8 (3)	N5—C30—H30B	109.321
S7—C22—N4	118.4 (3)	C31—C30—H30A	109.322
S8—C22—N4	118.8 (3)	C31—C30—H30B	109.321
N4—C23—C24	114.5 (4)	H30A—C30—H30B	107.964
C23—C24—C25	114.8 (4)	C30—C31—H31A	108.259
C24—C25—C26	114.4 (4)	C30—C31—H31B	108.272
C25—C26—C27	114.5 (4)	C32—C31—H31A	108.268
C26—C27—C28	113.5 (4)	C32—C31—H31B	108.263
N4—C28—C27	111.1 (4)	H31A—C31—H31B	107.388
S9—C29—S10	123.3 (2)	C31—C32—H32A	108.853
S9—C29—N5	118.9 (3)	C31—C32—H32B	108.856
S10—C29—N5	117.7 (3)	C33—C32—H32A	108.868
N5—C30—C31	111.5 (4)	C33—C32—H32B	108.857
C30—C31—C32	116.1 (5)	H32A—C32—H32B	107.714
C31—C32—C33	113.5 (4)	C32—C33—H33A	108.188
C32—C33—C34	116.4 (4)	C32—C33—H33B	108.191
C33—C34—C35	115.9 (4)	C34—C33—H33A	108.201
N5—C35—C34	115.1 (4)	C34—C33—H33B	108.204
N1—C2—H2A	109.037	H33A—C33—H33B	107.360
N1—C2—H2B	109.040	C33—C34—H34A	108.309
C3—C2—H2A	109.036	C33—C34—H34B	108.295
C3—C2—H2B	109.026	C35—C34—H34A	108.294
H2A—C2—H2B	107.815	C35—C34—H34B	108.291
C2—C3—H3A	108.402	H34A—C34—H34B	107.397
C2—C3—H3B	108.411	N5—C35—H35A	108.497
C4—C3—H3A	108.405	N5—C35—H35B	108.489
C4—C3—H3B	108.417	C34—C35—H35A	108.499
H3A—C3—H3B	107.470	C34—C35—H35B	108.512
C3—C4—H4A	108.830	H35A—C35—H35B	107.511
C3—C4—H4B	108.820		
			
Cu2—Br2—Cu6—S6	68.46 (3)	Cu2—S1—C1—S2	−102.52 (18)
Cu2—Br2—Cu6—S9	−139.63 (3)	Cu2—S1—C1—N1	80.1 (3)
Cu6—Br2—Cu2—S1	140.97 (4)	Cu1—S2—C1—S1	6.99 (17)
Cu6—Br2—Cu2—S5	−62.17 (3)	Cu1—S2—C1—N1	−175.7 (3)
Cu3—Br3—Cu7—S8	−69.04 (3)	Cu3—S2—C1—S1	99.05 (18)
Cu3—Br3—Cu7—S9	139.23 (4)	Cu3—S2—C1—N1	−83.6 (3)
Cu7—Br3—Cu3—Br1	−44.60 (2)	Cu1—S3—C8—S4	−7.11 (16)
Cu7—Br3—Cu3—S2	−149.31 (4)	Cu1—S3—C8—N2	174.8 (3)
Cu7—Br3—Cu3—S7	71.33 (3)	Cu4—S3—C8—S4	−103.43 (17)
Cu5—Br4—Cu9—S6	−70.08 (3)	Cu4—S3—C8—N2	78.5 (3)
Cu5—Br4—Cu9—S10	142.80 (4)	Cu1—S4—C8—S3	7.05 (16)
Cu9—Br4—Cu5—S4	−147.38 (4)	Cu1—S4—C8—N2	−174.9 (3)
Cu9—Br4—Cu5—S5	66.80 (4)	Cu5—S4—C8—S3	100.02 (17)
Cu4—Br5—Cu8—S8	65.76 (3)	Cu5—S4—C8—N2	−82.0 (3)
Cu4—Br5—Cu8—S10	−139.95 (4)	Cu2—S5—C15—S6	57.3 (3)
Cu8—Br5—Cu4—S3	140.59 (4)	Cu2—S5—C15—N3	−121.8 (3)
Cu8—Br5—Cu4—S7	−65.85 (3)	Cu5—S5—C15—S6	−38.0 (4)
S1—Cu1—S2—Cu3	−110.91 (4)	Cu5—S5—C15—N3	142.9 (3)
S1—Cu1—S2—C1	−5.06 (4)	Cu6—S6—C15—S5	−48.2 (3)
S2—Cu1—S1—Cu2	109.51 (5)	Cu6—S6—C15—N3	130.9 (3)
S2—Cu1—S1—C1	5.04 (4)	Cu9—S6—C15—S5	31.4 (4)
S1—Cu1—S4—Cu5	74.84 (4)	Cu9—S6—C15—N3	−149.4 (3)
S1—Cu1—S4—C8	178.51 (4)	Cu3—S7—C22—S8	−19.7 (4)
S4—Cu1—S1—Cu2	−72.42 (5)	Cu3—S7—C22—N4	161.4 (3)
S4—Cu1—S1—C1	−176.89 (4)	Cu4—S7—C22—S8	68.7 (3)
S2—Cu1—S3—Cu4	−73.27 (5)	Cu4—S7—C22—N4	−110.3 (3)
S2—Cu1—S3—C8	−176.80 (4)	Cu7—S8—C22—S7	19.6 (4)
S3—Cu1—S2—Cu3	72.60 (4)	Cu7—S8—C22—N4	−161.5 (3)
S3—Cu1—S2—C1	178.45 (4)	Cu8—S8—C22—S7	−65.8 (3)
S3—Cu1—S4—Cu5	−108.71 (4)	Cu8—S8—C22—N4	113.1 (3)
S3—Cu1—S4—C8	−5.04 (4)	Cu6—S9—C29—S10	−32.7 (3)
S4—Cu1—S3—Cu4	108.57 (5)	Cu6—S9—C29—N5	147.7 (3)
S4—Cu1—S3—C8	5.03 (4)	Cu7—S9—C29—S10	64.2 (3)
Br2—Cu2—S1—Cu1	−123.10 (4)	Cu7—S9—C29—N5	−115.3 (3)
Br2—Cu2—S1—C1	−36.28 (6)	Cu8—S10—C29—S9	−62.7 (3)
Br2—Cu2—S5—Cu5	124.46 (4)	Cu8—S10—C29—N5	116.9 (3)
Br2—Cu2—S5—C15	13.81 (6)	Cu9—S10—C29—S9	28.8 (4)
S1—Cu2—S5—Cu5	−76.56 (4)	Cu9—S10—C29—N5	−151.7 (3)
S1—Cu2—S5—C15	172.78 (4)	C1—N1—C2—C3	142.5 (4)
S5—Cu2—S1—Cu1	77.44 (5)	C2—N1—C1—S1	−1.7 (6)
S5—Cu2—S1—C1	164.26 (4)	C2—N1—C1—S2	−178.7 (3)
Br1—Cu3—S2—Cu1	20.96 (3)	C1—N1—C7—C6	−87.0 (4)
Br1—Cu3—S2—C1	−64.88 (5)	C7—N1—C1—S1	177.7 (3)
Br1—Cu3—S7—Cu4	−22.61 (3)	C7—N1—C1—S2	0.6 (5)
Br1—Cu3—S7—C22	75.36 (6)	C2—N1—C7—C6	92.4 (4)
Br3—Cu3—S2—Cu1	129.77 (4)	C7—N1—C2—C3	−36.9 (5)
Br3—Cu3—S2—C1	43.92 (7)	C8—N2—C9—C10	80.9 (5)
Br3—Cu3—S7—Cu4	−139.06 (4)	C9—N2—C8—S3	2.7 (5)
Br3—Cu3—S7—C22	−41.09 (8)	C9—N2—C8—S4	−175.1 (3)
S2—Cu3—S7—Cu4	80.56 (5)	C8—N2—C14—C13	−100.8 (4)
S2—Cu3—S7—C22	178.54 (6)	C14—N2—C8—S3	179.1 (3)
S7—Cu3—S2—Cu1	−85.86 (5)	C14—N2—C8—S4	1.3 (5)
S7—Cu3—S2—C1	−171.70 (5)	C9—N2—C14—C13	75.8 (4)
Br5—Cu4—S3—Cu1	−121.92 (4)	C14—N2—C9—C10	−95.8 (4)
Br5—Cu4—S3—C8	−34.94 (6)	C15—N3—C16—C17	88.8 (4)
Br5—Cu4—S7—Cu3	125.75 (3)	C16—N3—C15—S5	−0.1 (6)
Br5—Cu4—S7—C22	15.20 (5)	C16—N3—C15—S6	−179.3 (3)
S3—Cu4—S7—Cu3	−77.57 (4)	C15—N3—C21—C20	−78.5 (5)
S3—Cu4—S7—C22	171.88 (4)	C21—N3—C15—S5	170.2 (3)
S7—Cu4—S3—Cu1	82.50 (5)	C21—N3—C15—S6	−9.0 (6)
S7—Cu4—S3—C8	169.47 (4)	C16—N3—C21—C20	92.5 (4)
Br4—Cu5—S4—Cu1	126.53 (4)	C21—N3—C16—C17	−82.2 (5)
Br4—Cu5—S4—C8	40.90 (7)	C22—N4—C23—C24	143.7 (4)
Br4—Cu5—S5—Cu2	−131.24 (4)	C23—N4—C22—S7	−0.7 (6)
Br4—Cu5—S5—C15	−26.61 (8)	C23—N4—C22—S8	−179.7 (4)
S4—Cu5—S5—Cu2	79.11 (5)	C22—N4—C28—C27	−86.1 (5)
S4—Cu5—S5—C15	−176.26 (6)	C28—N4—C22—S7	177.5 (4)
S5—Cu5—S4—Cu1	−83.23 (5)	C28—N4—C22—S8	−1.5 (6)
S5—Cu5—S4—C8	−168.86 (5)	C23—N4—C28—C27	92.2 (5)
Br2—Cu6—S6—Cu9	−141.13 (3)	C28—N4—C23—C24	−34.6 (6)
Br2—Cu6—S6—C15	−33.28 (6)	C29—N5—C30—C31	96.8 (4)
Br2—Cu6—S9—Cu7	77.08 (4)	C30—N5—C29—S9	3.5 (6)
Br2—Cu6—S9—C29	−179.40 (4)	C30—N5—C29—S10	−176.1 (4)
S6—Cu6—S9—Cu7	−133.66 (5)	C29—N5—C35—C34	−84.5 (5)
S6—Cu6—S9—C29	−30.14 (7)	C35—N5—C29—S9	179.2 (4)
S9—Cu6—S6—Cu9	69.54 (5)	C35—N5—C29—S10	−0.4 (6)
S9—Cu6—S6—C15	177.39 (4)	C30—N5—C35—C34	91.5 (4)
Br3—Cu7—S8—Cu8	141.88 (4)	C35—N5—C30—C31	−79.3 (4)
Br3—Cu7—S8—C22	41.45 (8)	N1—C2—C3—C4	−44.5 (5)
Br3—Cu7—S9—Cu6	−77.95 (5)	C2—C3—C4—C5	88.1 (5)
Br3—Cu7—S9—C29	172.10 (4)	C3—C4—C5—C6	−67.0 (4)
S8—Cu7—S9—Cu6	129.28 (5)	C4—C5—C6—C7	52.6 (5)
S8—Cu7—S9—C29	19.33 (7)	C5—C6—C7—N1	−75.0 (5)
S9—Cu7—S8—Cu8	−66.64 (5)	N2—C9—C10—C11	40.9 (5)
S9—Cu7—S8—C22	−167.08 (6)	C9—C10—C11—C12	36.9 (5)
Br5—Cu8—S8—Cu7	−135.47 (3)	C10—C11—C12—C13	−85.7 (4)
Br5—Cu8—S8—C22	−21.58 (6)	C11—C12—C13—C14	68.9 (5)
Br5—Cu8—S10—Cu9	74.61 (4)	C12—C13—C14—N2	−50.2 (5)
Br5—Cu8—S10—C29	−173.83 (4)	N3—C16—C17—C18	54.9 (5)
S8—Cu8—S10—Cu9	−132.00 (4)	C16—C17—C18—C19	−67.9 (5)
S8—Cu8—S10—C29	−20.44 (6)	C17—C18—C19—C20	87.2 (5)
S10—Cu8—S8—Cu7	68.45 (5)	C18—C19—C20—C21	−45.3 (5)
S10—Cu8—S8—C22	−177.67 (4)	C19—C20—C21—N3	−31.7 (5)
Br4—Cu9—S6—Cu6	142.29 (3)	N4—C23—C24—C25	−45.9 (5)
Br4—Cu9—S6—C15	38.72 (8)	C23—C24—C25—C26	87.6 (5)
Br4—Cu9—S10—Cu8	−73.53 (5)	C24—C25—C26—C27	−66.5 (6)
Br4—Cu9—S10—C29	−174.03 (5)	C25—C26—C27—C28	52.9 (6)
S6—Cu9—S10—Cu8	139.82 (5)	C26—C27—C28—N4	−78.4 (5)
S6—Cu9—S10—C29	39.32 (8)	N5—C30—C31—C32	56.5 (5)
S10—Cu9—S6—Cu6	−70.91 (5)	C30—C31—C32—C33	−69.0 (5)
S10—Cu9—S6—C15	−174.48 (6)	C31—C32—C33—C34	82.9 (5)
Cu1—S1—C1—S2	−7.02 (17)	C32—C33—C34—C35	−40.4 (5)
Cu1—S1—C1—N1	175.6 (3)	C33—C34—C35—N5	−35.4 (5)

## References

[bb1] Cardell, D., Hogarth, G. & Faulkner, S. (2006). *Inorg. Chim. Acta*, **359**, 1321–1324.

[bb2] Golding, R. M., Rae, A. D. & Sulligoi, L. (1974). *Inorg. Chem.* **13**, 2499–2504.

[bb3] Hendrickson, A. R., Martin, R. L. & Taylor, D. (1975). *J. Chem. Soc. Chem. Commun.* pp. 843–844.

[bb4] Jian, F., Wang, Z., Bai, Z., You, X., Fun, H.-K., Chinnakali, K. & Razak, I. R. (1999). *Polyhedron*, **18**, 3401–3406.

[bb5] Liao, P. K., Fang, C. S., Edwards, A. J., Kahlal, S., Saillard, J. Y. & Liu, C. W. (2012). *Inorg. Chem.* **51**, 6577–6591. 10.1021/ic300135w 22663192

[bb6] Ngo, S. C., Banger, K. K., DelaRosa, M. J., Toscano, P. J. & Welch, J. T. (2003). *Polyhedron*, **22**, 1575–1583.

[bb7] Okubo, T., Kuwamoto, H., Kim, K. H., Hayami, S., Yamano, A., Shiro, M., Maekawa, M. & Kuroda-Sowa, T. (2011). *Inorg. Chem.* **50**, 2708–2710. 10.1021/ic101794u 21355562

[bb8] Okubo, T., Tanaka, N., Kim, K. H., Anma, H., Seki, S., Saeki, A., Maekawa, M. & Kuroda-Sowa, T. (2011). *Dalton Trans.* **40**, 2218–2224. 10.1039/c0dt01065k 21180735

[bb9] Okubo, T., Tanaka, N., Kim, K. H., Yone, H., Maekawa, M. & Kuroda-Sowa, T. (2010). *Inorg. Chem.* **49**, 3700–3702. 10.1021/ic1003098 20307080

[bb10] Rigaku (1995). *ABSCOR* Rigaku Corporation, Tokyo, Japan.

[bb11] Rigaku (2006). *RAPID-AUTO* Rigaku Corporation, Tokyo, Japan.

[bb12] Rigaku (2010). *CrystalStructure* Rigaku Corporation, Tokyo, Japan.

[bb13] Sheldrick, G. M. (2008). *Acta Cryst.* A**64**, 112–122. 10.1107/S0108767307043930 18156677

